# Editorial: Advances in ocular imaging and biometry

**DOI:** 10.3389/fmed.2022.1036038

**Published:** 2022-09-27

**Authors:** Hongxuan Zeng, Weining Zhu, Yuan Tan, Zhenzhen Liu

**Affiliations:** ^1^State Key Laboratory of Ophthalmology, Zhongshan Ophthalmic Center, Sun Yat-sen University, Guangzhou, China; ^2^Guangdong Provincial Key Laboratory of Ophthalmology and Visual Science, Guangzhou, China; ^3^Guangdong Provincial Clinical Research Center for Ocular Diseases, Guangzhou, China; ^4^Zhongshan Medical School, Sun Yat-sen University, Guangzhou, China

**Keywords:** ocular imaging, biometry, accuracy, measurement agreement, artificial intelligence

The eye is an organ of vision composed of a set of refractive elements, including cornea, aqueous humor, crystalline lens, and vitreous humor. These refractive elements are transparent in order to allow light to shine on the retina. Transparency of the refractive media allows us to directly observe these anatomical structures and internal blood vessels. Therefore, medical imaging is of great value in the diagnosis and treatment of ophthalmic diseases. The multifaceted and multilevel understanding of ocular anatomy and pathology has greatly improved clinicians' diagnosis and expanded ocular examination and diagnosis methods.

In addition to imaging devices applied in multiple medical disciplines, such as magnetic resonance imaging (MRI), ultrasonography, and electron computed tomography (CT), in the field of ophthalmology, there are special biomedical optical tools such as fundus angiography, optical coherence tomography (OCT), and ultrasound biomicroscopy (UBM), all of which allow us to obtain intuitive, high-resolution, and dynamic image data. At the same time, these preserved imaging data can make a great contribution to the future diagnosis of difficult diseases and deepen the understanding of ocular tissues. The interdisciplinary combination of cutting-edge technologies, such as artificial intelligence with medicine, has resulted in modern medical imaging becoming an emerging discipline.

This Research Topic, “*Advances in Ocular Imaging and Biometry*,” includes articles on recent advances in different types of imaging and biometry. We pay special attention to the current state of opinion, the accuracy of new biometric instruments, the efficiency novel technologies alternative to traditional ones, and the accuracy and consistency between different measurement instruments.

## Optical coherence tomography

Compared with other optical imaging systems, OCT has its own unique advantages, which bridges a gap between confocal microscopy and ultrasound imaging with a resolution of 1–15 μm and a penetration depth of 1–2 mm. The Fourier-domain OCT is divided into Spectral-domain OCT (SD-OCT) and Swept-source OCT (SS-OCT).

### Swept-source optical coherence tomography

Chen et al. investigated lens biometric parameters of congenital lens aberrations using CASIA2, a machine with a new technology of swept-source anterior segment optical coherence tomography (SS-ASOCT). CASIA2 was found to be a valuable option for *in vivo* crystallography of congenital lens aberrations, which can help accurately diagnose microphthalmia (MSP), lens warts (CL), and posterior lens cones (PL), providing enlightening insights into the pathogenesis of these diseases.

Huang et al. developed an algorithm to detect and quantify hyperreflectivity (HRD) on OCT in patients with diabetic macular edema (DME).

### Spectral-domain optical coherence tomography

Domínguez-Vicent et al. performed macular and optic disc volume measurements by separate and combined scanning schemes using the SD-OCT instrument: the Canon OCT HS-100. Macular and optic disc scans alone were found to have better reproducibility than the combined scan mode, but the two scan modes could not be used interchangeably due to extensive consistency limitations.

*In vivo* imaging of the retina and choroid using SD-OCT in guinea pigs and evaluation of its feasibility, concluding that OCT-based *in vivo* morphometric imaging of the guinea pig retina and choroid is feasible with acceptable intra-observer reproducibility and inter-observer reproducibility (Dong et al.).

Lin et al. used SD-OCT to identify interpretable OCT imaging biomarkers such as central subfield thickness (CST), macular fissure area (AMS) and photoreceptor defects in patients with X-linked retinopathies.

### Optical coherence tomography angiography

Arrigo et al. were able to identify two clinically distinct subtypes of macular neovascularization (MNV) by combining OCTA with conventional angiography: one with more exudation but less damage to the outer retinal structures, and the other with less secretion but causing irreversible anatomical damage.

Qian et al. found that wide-field SS-OCTA can more comprehensively assess choroidal retinal changes in patients with Govt-Koyanagi-Harada (VKH) disease.

Zhong et al. concluded that the retinal vascular system can be used as a surrogate for detecting microvascular injury and help evaluate patients with complete coronary artery occlusion (CTO), and that OCTA examination can be used to monitor the course of coronary artery disease by measuring the retinal vascular system in patients by OCTA.

## MRI

Liang et al. assessed spontaneous brain activity by rs-fMRI scanning and fractional amplitude of low frequency fluctuations (fALFF) methods, and concluded that high spontaneous activity in two brain regions may reflect the neuropathological mechanism of visual impairment in patients with high intraocular pressure (OH).

## Compare the results between different instruments

Ruan et al. evaluated the reproducibility of a new ultrasound biomicroscope, Insight 100, and its agreement with scanning source optical coherence chromatography imaging (CASIA2). The Insight 100 was found to obtain highly reproducible lens biometry *in vivo* with better signal penetration.

Chen et al. compared the measurement accuracy of the new optical biometer Pentacam AXL (Scheimpflug imaging with partial coherence interferometry) with that of the OA-2000 biometer (scanning source optical coherence laminar imaging and Placido disk topography) and found high agreement between the two devices to be clinically interchangeable for measuring most ocular parameters.

Du et al. compared vault measurements using scanning source OCT (IOLMasrer 700) and ocular segment OCT and found that the IOLMasrer 700 was able to measure the dome of the implanted lens with high accuracy and reproducibility, and observed good correlation and agreement between the IOLMaster 700 and AS-OCT.

The other articles in this section also have their own unique innovations. Whether it is collecting fundus images or building models, new types of instruments or techniques are used. These articles have broadened our thinking and provided more selectivity for diagnosis and treatment.

## Author contributions

HZ and ZL drafted the manuscript. WZ and YT provided revisions for the manuscript. All authors contributed to the article and approved the submitted version.

## Funding

This study was supported by the National Natural Science Foundation of China (81873675) and the Youth Project of State Key Laboratory of Ophthalmology (2022QN03 and 2021QN02).

## Conflict of interest

The authors declare that the research was conducted in the absence of any commercial or financial relationships that could be construed as a potential conflict of interest.

## Publisher's note

All claims expressed in this article are solely those of the authors and do not necessarily represent those of their affiliated organizations, or those of the publisher, the editors and the reviewers. Any product that may be evaluated in this article, or claim that may be made by its manufacturer, is not guaranteed or endorsed by the publisher.

